# Biomechanical Response of the Lower Extremity to Running-Induced Acute Fatigue: A Systematic Review

**DOI:** 10.3389/fphys.2021.646042

**Published:** 2021-08-27

**Authors:** Salil Apte, Gäelle Prigent, Thomas Stöggl, Aaron Martínez, Cory Snyder, Vincent Gremeaux-Bader, Kamiar Aminian

**Affiliations:** ^1^Laboratory of Movement Analysis and Measurement, École Polytechnique Fédérale de Lausanne, Lausanne, Switzerland; ^2^Department of Sport and Exercise Science, University of Salzburg, Salzburg, Austria; ^3^Institute of Sport Sciences, University of Lausanne,Lausanne, Switzerland; ^4^Swiss Olympic Medical Center, Sport Medicine Unit, Division of Physical Medicine and Rehabilitation, Lausanne University Hospital, Lausanne, Switzerland

**Keywords:** fatigue research, running, biomechanics, systematic review, wearable sensors, functional tests

## Abstract

**Objective:** To investigate (i) typical protocols used in research on biomechanical response to running-induced fatigue, (ii) the effect of sport-induced acute fatigue on the biomechanics of running and functional tests, and (iii) the consistency of analyzed parameter trends across different protocols.

**Methods:** Scopus, Web of Science, Pubmed, and IEEE databases were searched using terms identified with the Population, Interest and Context (PiCo) framework. Studies were screened following the Preferred Reporting Items for Systematic Reviews and Meta-Analyses (PRISMA) guidelines and appraised using the methodological index for non-randomized studies MINORS scale. Only experimental studies with at least 10 participants, which evaluated fatigue during and immediately after the fatiguing run were included. Each study was summarized to record information about the protocol and parameter trends. Summary trends were computed for each parameter based on the results found in individual studies.

**Results:** Of the 68 included studies, most were based on in-lab (77.9%) protocols, endpoint measurements (75%), stationary measurement systems (76.5%), and treadmill environment (54.4%) for running. From the 42 parameters identified in response to acute fatigue, flight time, contact time, knee flexion angle at initial contact, trunk flexion angle, peak tibial acceleration, CoP velocity during balance test showed an increasing behavior and cadence, vertical stiffness, knee extension force during MVC, maximum vertical ground reaction forces, and CMJ height showed a decreasing trend across different fatigue protocols.

**Conclusion:** This review presents evidence that running-induced acute fatigue influences almost all the included biomechanical parameters, with crucial influence from the exercise intensity and the testing environment. Results indicate an important gap in literature caused by the lack of field studies with continuous measurement during outdoor running activities. To address this gap, we propose recommendations for the use of wearable inertial sensors.

## Introduction

Appropriate management of acute fatigue, resulting from a training stimulus and aimed at triggering positive adaptation, is essential to optimize the benefits of a training program to athletes and reduce their risk of injury (Kellmann et al., 2018). In this context, acute fatigue refers to the onset of fatigue occurring concurrently with the training activity, with its influence measured during and/or within 30 min after the activity. Fatigue is a complex multi-factorial phenomenon, characterized by a drop in work capacity and the inability to generate the requisite muscular force to sustain simple or more complex tasks (Enoka and Duchateau, 2008; Taylor et al., 2016). The investigation of fatigue development mechanisms is a complex task, and surrogate measures of fatigue, such as self-reported score and changes in neuromuscular function, biomechanical parameters, and physiological processes (Taylor et al., 2012; Thorpe et al., 2016). Physiological responses are generally evaluated *via* heart rate monitoring, blood lactate, near-infrared spectroscopy, gas exchange measurements, etc., while rating of perceived exertion (RPE) and visual analog scales (VASs) are used to measure subjective feeling of fatigue (Thorpe et al., 2017). Neuromuscular functions and maximal force production capacity are normally tested with functional tests such as vertical jump tests, balance, and maximum voluntary contraction tests, using performance metrics such as maximal jump height, center of pressure movement, and maximal knee flexion torque (Thorpe et al., 2017). Finally, motion capture systems, force plates, and video analyses are generally utilized to analyze biomechanical changes (Thorpe et al., 2017). Recently, body-worn inertial measurement units (IMU), global navigation satellite systems (GNSSs), and pressure sensor-based insoles have been used for measuring biomechanical changes in addition to laboratory-based optical motion capture systems, since the former enable measurement in the field (Strohrmann et al., 2012; Buckley et al., 2017).

In this study, we examine the lower extremity biomechanical response as a surrogate measure for sport-induced acute fatigue. Biomechanical response is altered due to acute fatigue and, thus, it is of interest for re-measurement during/after training interventions to investigate the influence of fatigue (Paquette et al., 2020) Kinematics, kinetics, and muscle activity of the leg during running and the spatiotemporal gait parameters comprise the lower extremity biomechanical response, in addition to the aforementioned functional tests. Lower extremity injuries represent the most frequent injuries in sports (Nicholl et al., 1995; Emery and Pasanen, 2019), especially in those with high participation, such as athletics (Alonso et al., 2010). Since biomechanical changes are activity-specific and context dependent, the selection of relevant sporting activities is crucial. Running constitutes an important part of in-match activity and training regimens for athletics (Gamble, 2013), and training factors, such as high accelerations and large absolute training loads leading to repeated high acute fatigue states, represent a major risk factor for overuse injuries, especially in endurance running (Mizrahi et al., 2000a; Clansey et al., 2012; Warden et al., 2014; Dempster et al., 2021). Thus, this review is focused on acute fatigue induced by running activities. Examples of such running activities are events such as marathon, half-marathon, and trail running, and track-based and treadmill-based protocols such as repeated-sprints and incremental speed tests, etc. Improved comprehension of the influence of fatigue on the biomechanical changes during running and the functional tests involving the lower extremity neuromuscular response could enable improved training load prescriptions and injury risk management.

The current literature on the influence of fatigue regarding running gait parameters shows conflicting results. For example, a study showed a decrease in contact time after fatigue (Morin et al., 2011a), while another study showed no change (Morin et al., 2011b); one reported a reduction in peak knee flexion angle during stance phase (Chan-Roper et al., 2012), whereas another reported an increase (Jewell et al., 2017). The tasks used to produce fatigue varied considerably, ranging from studies on medium-intensity high-volume activities such as ultra-marathons (Morin et al., 2011b) and 24-h treadmill runs (Morin et al., 2011a) to severe-intensity intermittent activities such as repeated sprints (Johnston et al., 2015) or soccer matches (Matthews et al., 2017). Previous reviews (Giandolini et al., 2016; Winter et al., 2017) have tried to address these conflicting results on the influence of fatigue on running. However, the former of these studies included a small study sample, only considered prolonged running, and did not categorize the level of fatigue; while the latter study was not a systematic review and was more focused on graded running and its effects on physiological metrics. Both reviews did not present summary trends or comment on the sensor systems used for measurement. Thus, we are not aware of a review focusing on the influence of acute fatigue on the biomechanics of running and functional tests (as described above), which takes in account the nature of fatiguing activity and the measurement environment, and synthesizes evidence regarding possible trends for these parameters.

To address this problem, we defined the primary research question: “What is the effect of sport-induced acute fatigue on the biomechanics of running and functional tests?” Secondary questions were designed to understand the dominant biomechanical metrics used in fatigue research while investigating the consistency of their behavior across studies, and to explore the influence of fatiguing protocols. The scope of this review is limited to experimental research aimed at investigating lower extremity biomechanical response in healthy adults and published from 1990 to 2021. It only includes studies that used running as an activity to induce acute fatigue and analyzed it with non-invasive methods during the activity and/or immediately post-activity.

## Methods

### Search Strategy and Sources

The search strategy was based on the Population, Interest and Context (PICo) framework, with the goal of locating studies that explicitly report the experience of fatigue in healthy adults participating in sport activities (Mamédio et al., 2007). The search terms for each of these three categories were combined with a Boolean “AND” (Table 1). 63 search items excluding irrelevant publication types were combined with a Boolean “AND NOT.” Scopus, Web of Science, Pubmed, and IEEE databases were searched for papers published from 1990 to 2021 in English language.

**Table 1:** Details of the PiCO strategy used to conceptualize search terms.

**Population**	**Interest**	**Context**
Healthy adults doing sports Elite/non-elite	The experience of fatigue	Sports activities: running
“healthy” OR “athletes” OR “players” OR “sportperson” OR “runners”	“fatigue” OR “exertion” OR “exhaustion” OR “prolonged” OR “marathon” OR “ultramarathon” OR “long distance”	“run” OR “running” OR “endurance” OR “prolonged” OR “long distance” AND (“wearable” OR “sensors” OR “measure” OR “measurements” OR “reporting” OR “assess” OR “evaluate” OR “investigate” OR “collect” OR “collected”)

*The following terms were used with a “AND NOT” to exclude them: “supplement” OR “supplementation” OR “nutrition” OR “diet” OR “therapy” OR “doping” OR “pregnancy” OR “patients” OR “junior” OR “adolescent” OR “ingestion” OR “accident” OR “compression garments” OR “age” OR “animals” OR “immersion” OR “food” OR “disease” OR “epidemiology” OR “fracture” OR “stimulation” OR “dogs” OR “horses” OR “rehabilitation” OR “treatment” OR “concussion” OR “kids” OR “teenagers” OR “military” OR “obese” OR “obesity” OR “weight loss” OR “music” OR “swimming” OR “basketball” OR “rowing” OR “handball” OR “softball” OR “volleyball” OR “badminton” OR “tennis” OR “ice hockey” OR “skiing” OR “boxing” OR “cricket” OR “wrestling” OR “golf” OR “weightlifting” OR “martial art” OR “climbing” OR “gymnastics” OR “kayaking” OR “fencing” OR “shooting” OR “diving” OR “diesel” OR “gas” OR “engine” OR “cycling” OR “football” OR “soccer” OR “rugby” OR “ultramarathon”*.

### Eligibility Criteria

Following the Preferred Reporting Items for Systematic Reviews and Meta-Analyses (PRISMA) method (Page et al., 2021), studies obtained from the aforementioned databases were screened using the criteria mentioned below. If all the relevant information to exclude an article was available in the abstract, it was excluded at this stage. If not, the full text of the articles was screened for compliance. We summarized the parameter trends from individual studies based on significant results, and thus wanted to include publications having a reasonable statistical power. However, there is no consensus on the exact number of participants, as the sample size should be estimated based on the expected power and effect size. Furthermore, a higher cut-off for the number of participants would lead to a larger number of studies being excluded. Thus, we decided that a cut-off of 10 participants is an appropriate compromise between statistical power of the reported trends and publication exclusion criteria. In addition, one of the aims behind this study was to understand the evolution of fatigue measurement protocols over the recent decades, especially regarding the use of wearable sensors and conducting field measurements. Since the use of wearable sensors was limited before 1990, we decided to limit the scope to studies conducted onwards of 1990. For detailed screening criteria, please refer to Appendix A1 in Supplementary Material 3.

Inclusion criteria:

- Investigation of acute fatigue induced by running activities (as primary or secondary outcome) using noninvasive methods.
- A study population of at least 10 healthy adults (between 18 and 65 years old) engaged in sports activities.
- Original experiment-based research (systematic review/review/meta-analysis excluded).
- Clear description of the nature of activity, measurement conditions, and sensors used for measurements.
- Measurement of the effect of fatigue on biosignals during or before/after sporting activity.
- Measurement of the effect of fatigue on biosignals within 30 min after sporting activity and description of the measurement outcomes with respect to the last training or event.

Exclusion criteria:

- Studies that investigate neither running biomechanics nor functional test parameters.
- Studies that focus only on physiological responses (brain electrical activity (EEG), electrocardiogram (ECG) or respiration) of fatiguing exercises.
- Studies that only consider biochemical parameters such as lactate, creatine kinase, and cortisol, or questionnaires to assess the effect of sport-related fatigue, without using any additional sensors.
- Focus was on the evaluation of psychological effects of sport on mental health.
- Sole investigation of recovery time or training program after fractures, concussion, or any other injuries related to sport.
- Analysis of the effects of various therapies to reduce fatigue.
- Investigation of the influence of specific environmental conditions or performance-enhancing substances on fatigue or for training.
- Fatigue protocols based on the use of specific exercises, such as repetitive movements or strength training, instead of sporting activities.

### Study Classification and Data Extraction

The methodological quality of the selected studies was appraised quantitatively using the validated “methodological index for non-randomized studies” (MINORS) scale (Slim et al., 2003). The items (see Appendix A2 in Supplementary Material 3) were scored zero (not reported), one (reported but inadequate), or two (reported and adequate). The total score was normalized by the maximum possible score to obtain a final value between zero and one. The score of each study was used as a weight index for computing the general trends for each extracted parameter. Details of this method can be found in Section Data Synthesis and Appendix A2 in Supplementary Material 3.

Each study was summarized by two authors to record information about the participant demographic, the study protocol, and the reference methods to assess fatigue. Following data were extracted from each study:

- A. Exercise intensity: While the level of fatigue is difficult to quantify (Enoka and Duchateau, 2008), it is important to state the level of fatigue reached by the athletes. Thus, the intensity of the fatiguing activity was graded into four categories, based on the critical power model (Morton, 2006): (i) moderate—can be continued more than 1 h, below aerobic threshold, typically in a range of 65–75% of the maximal oxygen uptake (VO_2_max); (ii) heavy—can be performed up to 1 h, lactate increase, between aerobic and anaerobic thresholds, in a range of 80–90% VO_2_max; (iii) severe—which is tolerable for up to 30 min, no steady state of VO_2_, muscle metabolic and blood acid-base responses, above anaerobic threshold; and (iv) high-intensity intermittent—repetitive efforts such as repetitive sprints or interval runs. It is hereafter referred to as intermittent for the sake of brevity.
- B. Reference: Criteria used for ascertaining the exercise intensity and designing the fatigue protocol. Examples of these include the measurement of VO_2_max, blood lactate, heart rate reserve, and questionnaires. In the absence of any information about these methods, nature of competitive activities such as marathons or soccer matches was recorded.
- C. Environment: The measurement environment (laboratory or field). Regarding running biomechanics, treadmill and overground evaluations of fatigue were analyzed separately, as the biomechanical response to treadmill running may differ from overground running (Van Hooren et al., 2020).
- D. Timing: The timing of the data collection to assess fatigue—continuously during protocol, intermittently, or at the endpoints i.e., the beginning/end or before/after the fatigue protocol;
- E. Measurement system: Specifications of the measurement systems in terms of usability, whether they are wearable or stationary;
- F. Parameters: The parameters used to assess the effects of the fatigue activity and their category (see Sections Parameter Definition and Data Synthesis). For every parameter, we noted whether it increased or decreased in response to acute fatigue or did not change significantly.

### Parameter Definition

We extracted the list of parameters used to assess fatigue and their respective qualitative trends, whether they increase, decrease, or do not change. These parameters were classified into five categories: spatiotemporal, kinetic, kinematic, functional test, and muscle activity. The first three categories, i.e., the spatiotemporal, kinetic and kinematic parameters, are directly relevant to running biomechanics and, thus, are extracted only from studies that investigated the influence of fatigue on running. The spatiotemporal (ST) parameters are derived from basic variables reflecting the spatiality and temporality of foot-based placements; they contain cadence, contact time, flight time, stride length, step width, etc. The kinematic (KM) category refers to the positions, angles, velocities and accelerations of body segments and joints during run. The kinetic (KT) category describes the joint torques, forces, stiffness, and ground impact aspects of running mechanics. The muscle activity (MA) parameters included in this review comprise the electrical activity measured using electromyography (EMG). Finally, functional test (FT) refers to the set of metrics used to analyze jump tests, maximum voluntary contraction (MVC), balance, and walking tests. These functional tests are generally used to understand the influence of sport-induced fatigue on neuromuscular function. Unlike the previous three categories, extraction of parameters linked to the MA and FT categories was not limited to studies investigating running biomechanics. Since most of the included parameters are well-known in fields of sport science; definitions of those parameters are not explained in this study. Description of EMG metrics and complex parameters, such as local dynamic stability (LDS) coefficients, are available in Appendix A3 in Supplementary Material 3.

### Data Synthesis

We created four subgroups in order to take into account the intensity of the fatiguing task (moderate, heavy, severe, or intermittent). For the running biomechanics, we further created two subgroups for treadmill and overground environments. This led to eight subgroups for the ST, KM, and KT categories, and four subgroups for FT and MA. A parameter was included in data synthesis only if, at least, one of the subgroups had a total number of participants >30, all studies merged. We estimated that a threshold of 30 participants might correspond to three studies with at least 10 participants or one study including more than 30 participants. This allowed us to compute a meaningful median value even if two of those three studies had opposing trends and, thus, the obtained summary trends were also meaningful.

The first part of the data synthesis was to collate the number of studies pertaining to the general information from the protocol referring to information A to E in Section Study Classification and Data Extraction. The second part was the computation of summary trends for the list of parameters extracted to assess fatigue. Median (MED) and median absolute deviation (MAD) were utilized for this purpose (see Appendix A4 in Supplementary Material 3), since these are non-parametric and robust metrics. Parameters with MAD value >0.5 were considered to have no trend, i.e., no consistency across studies. MAD lower than 0.1 indicated agreement across studies and was characterized as “clear decrease” if MED was negative, “non-significant change” if MED was equal to zero, and “clear increase” if MED was positive. For 0.1 < MAD < 0.5, the trends were characterized as “partial decrease,” “non-significant change,” or “partial increase.”

## Results

### Study Selection

**Figure 1:**
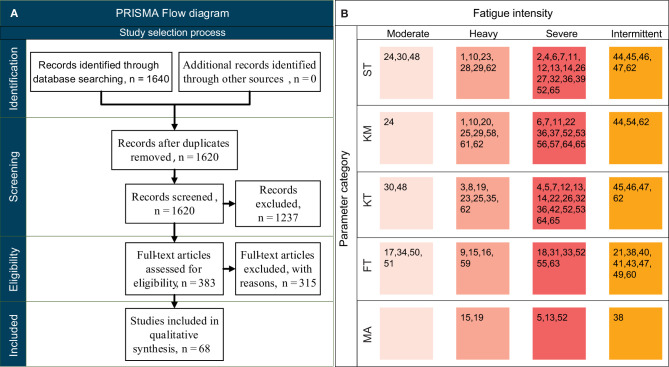
**(A)** PRISMA flow chart for study selection, adapted from Page et al. (2021). **(B)** References for the included 68 studies (1. Siler and Martin, 1991, 2. Verkerke et al., 1998, 3. Voloshin et al., 1998, 4. Willson and Kernozek, 1999, 5. Mizrahi et al., 2000a, 6. Mizrahi et al., 2000b, 7. Derrick et al., 2002, 8. Dutto and Smith, 2002, 9. Gómez et al., 2002, 10. Avogadro et al., 2003; 11. Borrani et al., 2003, 12. Mercer et al., 2003, 13. Weist et al., 2004, 14. Gerlach et al., 2005, 15. Racinais et al., 2007, 16. Bisiaux and Moretto, 2008, 17. Nagel et al., 2008, 18. Strang et al., 2008, 19. Wu et al., 2008, 20. Dierks et al., 2010, 21. Perrey et al., 2010, 22. Abt et al., 2011, 23. Alfuth and Rosenbaum, 2011, 24. Chan-Roper et al., 2012; 25. Clansey et al., 2012, 26. Clansey et al., 2016, 27. Hayes and Caplan, 2012, 28. Stirling et al., 2012, 29. Strohrmann et al., 2012, 30. Willems et al., 2012, 31. Dittrich et al., 2013, 32. Rabita et al., 2013, 33. Steib et al., 2013, 34. Easthope et al., 2014, 35. Garcia-Perez et al., 2014, 36. Hanley and Mohan, 2014, 37. Koblbauer et al., 2014, 38. Timmins et al., 2014; 39. Ammann and Wyss, 2015, 40. Goodall et al., 2015, 41. Johnston et al., 2015, 42. Anbarian and Esmaeili, 2016, 43. García-Pinillos et al., 2016a, 44. García-Pinillos et al., 2016b, 45. Girard et al., 2016, 46. Girard et al., 2017a, 47. Girard et al., 2017b, 48. Rosenbaum et al., 2016, 49. Rosso et al., 2016, 50. Rousanoglou et al., 2016, 51. Anna et al., 2017, 52. Jewell et al., 2017, 53. Radzak et al., 2017; 54. Bailey et al., 2018b, 55. Hamacher et al., 2018, 56. Hoenig et al., 2018, 57. Maas et al., 2018, 58. Mo and Chow, 2018, 59. Ribeiro et al., 2018, 60. Sánchez-Sánchez et al., 2018, 61. Bovalino et al., 2020, 62. Riazati et al., 2020, 63. Yu et al., 2020, 64. Yu et al., 2021, 65. Möhler et al., 2021), presented according to the fatigue intensity and the parameter category, where ST, spatiotemporal; KM, kinematic; KT, kinetic; FT, functional test; MA, muscle activity parameters. Studies that utilized machine-learning approaches (66. Eskofier et al., 2012, 67. Buckley et al., 2017, 68. Op De Beeck et al., 2018) and considered only statistical features in place of traditional metrics are not included in the table as they do not fit into any of the five parameter categories.

The literature search produced 1,640 records, which were screened using the process suggested in the PRISMA statement (Page et al., 2021) (Figure 1A). After removing 20 duplicates, abstracts of the remaining 1,620 studies were screened using the criteria described in Section Eligibility Criteria, resulting in 1,237 records being excluded. The full text of the remaining 383 records was assessed for eligibility, and 68 studies were included for the final evidence synthesis. The relevant parameters were extracted and classified using the categories defined in Section Parameter Definition (references for each are shown in Figure 1B). Detailed summary of the selected studies can be found in Supplementary Material 1.

### Characteristics of Selected Literature

#### Nature of Activities

**Figure 2:**
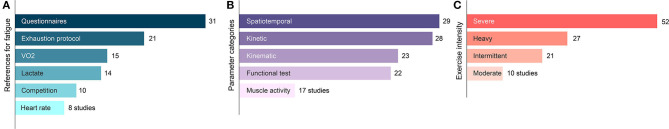
Number of studies investigating the different aspects of a fatigue research protocol **(A)**. Reference methods used to ascertain the fatigue intensity; **(B)** Parameter categories studied by the included protocols; **(C)** Exercise intensity investigated.

Most of the selected studies involved between 11 and 20 participants (58.8%), with only 12 studies testing more than 30 subjects (17.6%) and with a median MINORS index of 0.75. Detailed score for all 68 studies is presented in Supplementary Material 1. The number of participants ranged from 10 to 459, with a median (MAD) of 20 (±8) participants. The participants were a mixture of professional, semi-professional, and amateur athletes. While the exact definition of fatigue is not typically stated, six different methods (Figure 2A) were commonly used to investigate the level of fatigue. Questionnaires like the RPE and VAS were the most commonly used reference (31 studies), followed by custom-designed protocols (VO_2_max attainment, exhaustion protocols) to justify fatigue (21 studies), and blood lactate measurements (14 studies). The least used methods were based on heart rate and competitive racing events.

The exercise intensity was predominantly severe (52 studies), with protocols such as running until exhaustion (Figure 2). Heavy protocols represented 27 studies, while endurance-running activities, classified as moderate, were less commonly included (10 studies). Intermittent protocols, such as repeated sprints and high-intensity interval running, constituted 21 studies. Majority of the studies investigated spatiotemporal and kinetic parameters, with muscle activity being the least studied parameter group.

#### Nature of Measurement Environment

**Figure 3:**
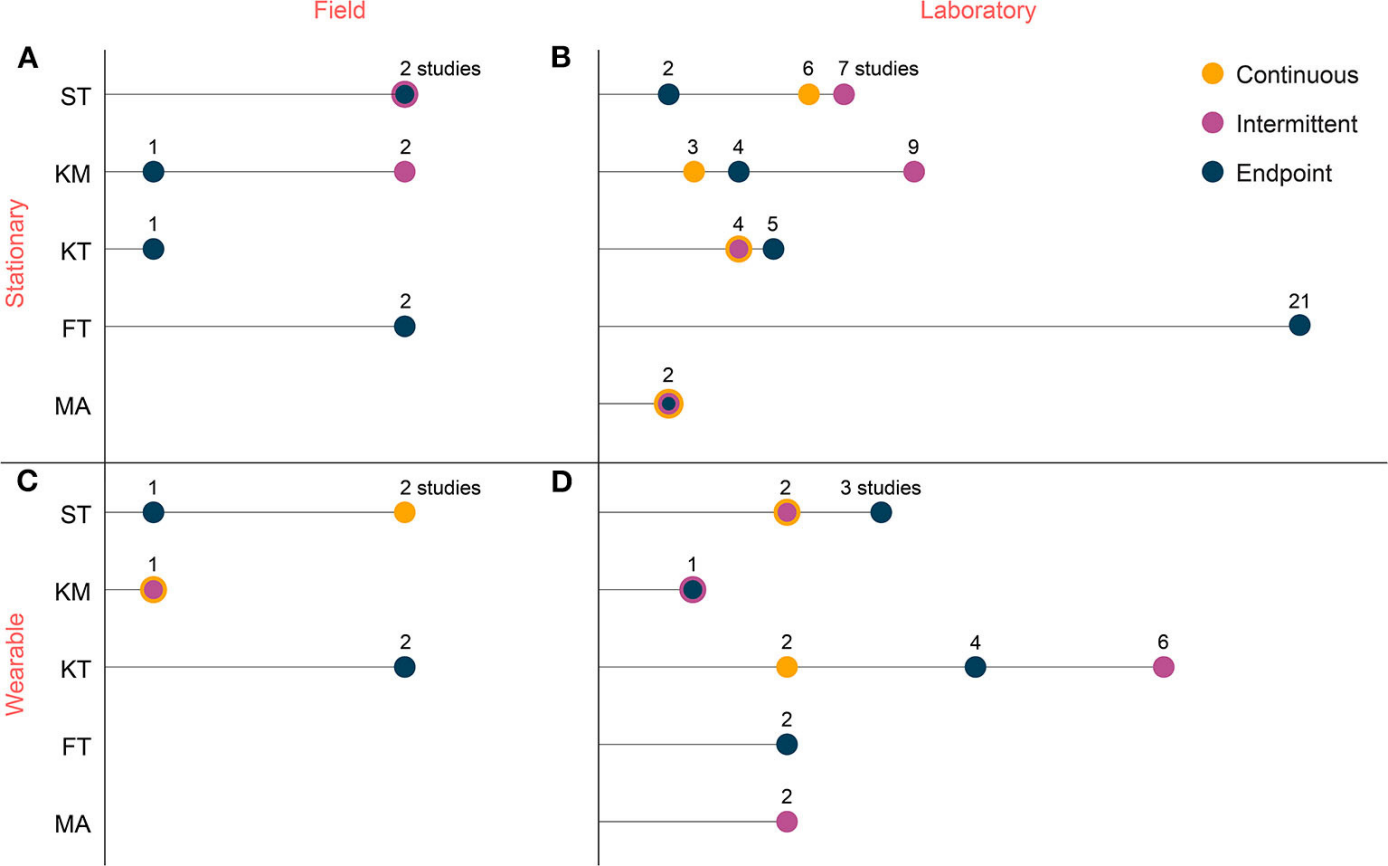
Number of studies per parameters category grouped in terms of the timing of the measurement (continuous, intermittent, and endpoint), sensors (wearable vs. stationary), and location (field vs. laboratory). **(A)** Field and stationary, **(B)** laboratory and stationary, **(C)** field and wearable, and **(D)** laboratory and wearable. The four sub-figures do not necessarily have the same scale on the x-axis. ST, gait spatiotemporal; KM, kinematics; KT, kinetics; FT, functional test; MA, muscle activity.

**Figure 4:**
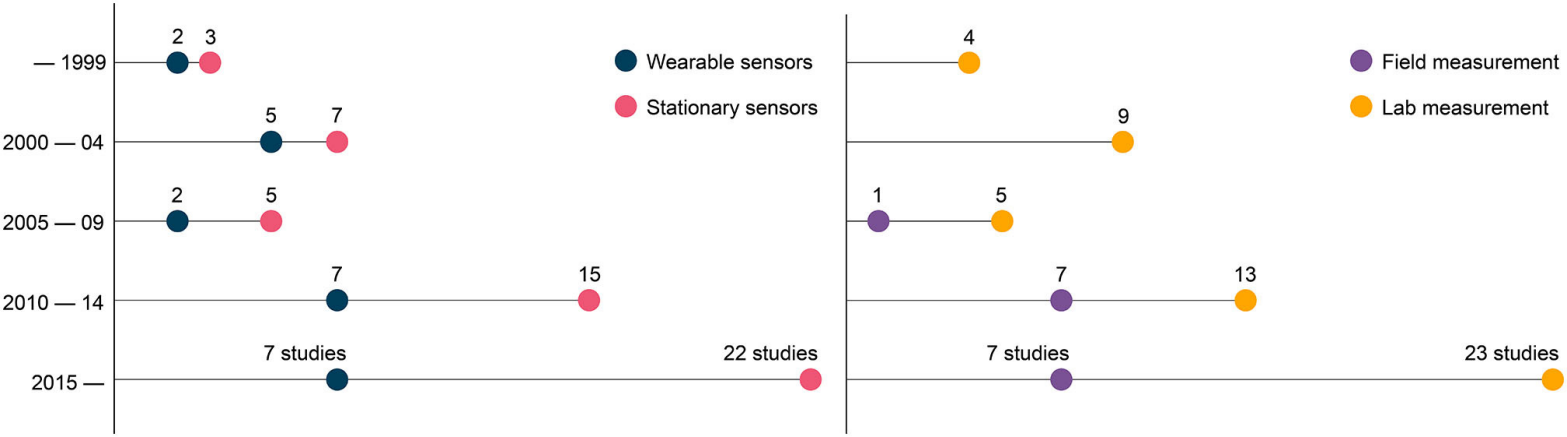
Number of studies utilizing wearable and/or stationary measurement systems and conducting research in lab and/or in field. The number of studies has increased drastically after 2010, yet the number of field studies and of those using wearable sensors has remained low.

We classified the studies in each of the five parameter categories based on their measurement system (stationary vs. wearable) and measurement environment (lab vs. field). As shown in Figure 3, measurements in the studied literature were mainly performed in-laboratory (77.9%) and typically with stationary measurement systems (76.5%) such as optical motion capture, instrumented treadmill, or force plates. Few studies used wearable sensors such as IMU, GNSS, pressure sensor-based insoles, heart rate telemetry, wireless EMG, or portable gas exchange systems allowing field measurements, which is in agreement with the low percentage of in-field protocols (22.1%). The number of studies analyzing sport-induced fatigue almost doubled after 2010 (Figure 4), with a similar increase for studies using wearable sensors. However, the ratio between the number of studies with stationary and wearable systems hardly changed over time. An important aspect of the protocol is the timing employed to perform the measurements. Most (75%) of the studies included used endpoint assessments by collecting data before and after the fatiguing exercise, followed by intermittent and continuous assessments respectively.

Most of the studies (71.3%) performed fatigue assessment in laboratory settings using stationary systems (Figure 3B). Furthermore, functional tests were usually conducted before/after the fatigue protocol (endpoint) in both laboratory and field environments (Figures 3A,B), typically with stationary measurement systems. Wearable sensors, despite their potential for field use, were mainly used in laboratory (Figure 3D) for assessing ST, KT, or MA parameters continuously and intermittently. Out of the 68 studies, only 4 studies assessed ST, KM, or KT parameters continuously or intermittently in the field using wearable sensors (Figure 3C).

#### Parameters for Analysis

The systematic extraction of parameters used by the studies to assess fatigue produced a list of 229 metrics. After removing parameters extracted on <30 participants (all studies merged), we obtained the final list of 42 parameters shown in Table 2. Supplementary Material 1 contains the complete list of metrics.

**Table 2:** Parameters trends across different protocols.

	**Parameters**	**Treadmill**				**Overground**				
		**M**	**H**	**S**	**I**	**M**	**H**	**S**	**I**	**S/T**
ST	Cadence (steps/min)	—	↓↓	**↓**	**↓*↓***	**↓**	**I**	**I**	—	14/22
	Contact time (ms)	—	**↑*↑***	**↑**	**↑*↑***	**↑*↑***	**I**	**↑*↑***	**↑*↑***	17/20
	Flight time (ms)	—	**I**	**↑*↑***	**↑**	—	—	**I**	**↑**	7/9
	Stride length (m)	—	**↑**	**↓*↑***	**↔↔**	**↓*↓***	**I**	**I**	**I**	8/16
KT	Max GRF (N)	—	**I**	**↓*↓***	**I**	**I**	**I**	**↓*↓***	—	7/10
	Loading rate (N/s)	—	—	**↓*↑***	**I**	—	**I**	—	—	4/5
	Peak pressure—metatarsal (Pa)	—	**I**	**↑**	—	**↑*↑***	**I**	**↓*↑***	—	6/10
	FTI—heel (N)	—	—	**↓**	—	**↔↔**	**I**	**↔↔**	—	4/8
	FTI—midfoot (N)	—	—	**↑*↑***	—	**↓*↑***	—	**↑**	—	4/6
	FTI—metatarsal (N)	—	—	**↔↔**	—	**↑**	—	**↑**	—	5/11
	FTI—toes (N)	—	—	**↑**	—	**↓**	—	**↓*↑***	—	4/7
	Peak tibial acceleration (PTA)	—	**↑**	**↑**	—	—	**↑**	—	—	2/8
	Peak head acceleration (PHA)	—	**I**	**↔↔**	—	—	**↑**	—	—	3/6
	Vertical stiffness (N/m)	—	**↓*↓***	—	**↓*↓***	—	**I**	**↓**	—	8/9
	Leg stiffness (N/m)	—	—	—	**↓**	—	—	**↓*↓***	—	6/8
	Mechanical work (J)	—	—	**↔↔**	—	—	—	—	—	1/3
KM	Knee—max. flexion angle (swing)	—	**↑*↑***	**↔↔**	—	**↓*↓***	**I**	—	—	5/7
	Knee—flexion angle at IC	—	—	**↑*↑***	—	—	—	—	**I**	2/3
	Knee—ROM f/e angle (stance)	—	**↔↔**	**↑*↑***	**I**	—	—	—	—	2/6
	Hip—ROM f/e angle (stance)	—	**I**	**↑**	**I**	—	—	—	—	$\frac{3}{4}$
	Hip—max adduction angle	—	**↑**	**↑**	**I**	—	**I**	—	—	3/6
	Ankle—PF angle at IC	—	—	**↓**	—	—	**↓*↓***	—	**I**	3/5
	Trunk—max flexion angle	—	**↑*↑***	**↑*↑***	—	—	**I**	—	—	5/6
	Pelvis—anterior tilt	—	—	**↑*↑***	—	—	—	—	—	2/2
	Pelvis—rotation ROM	—	—	**↑*↑***	—	—	—	—	—	2/2
	Pelvis and thorax—LDS	—	—	—	—	—	—	**↑*↑***	—	1/1
		**M**		**H**		**S**		**I**		**N**
FT	CMJ height	**↓*↓***		—		**I**		**↓**		5/7
	SJ height	—		—		—		**↓**		$\frac{1}{2}$
	DJ stabilization time	—		—		**↑*↑***		—		1/1
	Balance—CoP velocity	—		—		**↑*↑***		**↑**		2/3
	MVC force (knee extension)	—		**I**		**↓**		**↓*↓***		6/7
	Sprint completion time	—		—		—		**↑*↑***		3/3
	Walking—contact time	**↓*↓***		—		—		—		1/1
	Walking—peak pressure toes	**↓*↓***		—		—		—		1/1
	Walking—total foot contact area	**↓*↓***		—		—		—		1/1
	Walking—forefoot loading imp.	**↓*↓***		—		—		—		1/1
	Gait LDS—dual task walking	—		—		**↑*↑***		—		1/1
MA	iEMGquadricep	—		**I**		**↓*↑***		**I**		4/5
	iEMG hamstring	—		**I**		**↓**		—		1/3
	iEMG calf	—		**I**		**↓**		—		3/5
	iEMG shin	—		**I**		**↔↔**		—		2/4
	MF Calf	—		—		**↔↔**		—		1/3

*For a parameter, S, number of studies with significant results; and T, total number of studies measuring that parameter. The arrows represent following trends; 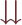, clear decrease; 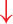, partial decrease; 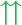, clear increase; 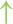, partial increase; 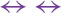 ↔, non-significant change, 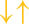
↓↑, no trend; and I, insufficient participants (<30).*

*For the parameters, GRF, ground reaction force; FTI, force-time integral; IC, initial contact; ROM, range of motion; f/e, flexion/extension;, LDS, local dynamic stability; CMJ, countermovement jump; SJ, squat jump; DJ, drop jump; CoP, center of pressure; MVC, maximum voluntary contraction; iEMG, integration over the EMG signal; MF, median frequency. Parameter trends, with M, moderate; H, heavy; S, severe; I, intermittent exercise intensities*.

*For actual median and MAD values, please refer to Supplementary Material 1*.

### Parameter Trends

Of the four gait ST parameters considered, cadence was measured most often and step length the least. Apart from cadence, contact time and flight time, which presented reliable and consistent trends (increase) across all different conditions, the trends obtained for stride length (Table 2) were dependent on the fatigue protocols and the running environments.

Of the 12 parameters in the KT category, only maximum ground reaction force (Max GRF), vertical stiffness, and leg stiffness presented a consistent trend (decrease) across the different exercise intensities and running environments. Max GRF was also the most commonly used metric (12 studies), followed by vertical and leg stiffness (10 studies each). Peak tibial and head acceleration (PTA and PHA) are two parameters extracted from body-worn accelerometers; PHA showed different trends between overground and treadmill running environments.

Within the 10 KM parameters investigated, LDS was the least studied (one study) and the peak knee flexion angle the most studied (seven studies). Peak knee flexion angle at initial contact (IC) and peak trunk flexion showed a clear increase for severe intensity during treadmill running, while ankle plantarflexion angle IC presented a clear decrease. Pelvic and thoracic LDS parameters are documented only for overground running with severe intensity and pelvis rotation range of motion (ROM) and anterior tilt for treadmill running with severe intensity; all present a clear increase because of fatigue.

Functional tests were always performed before and after the fatiguing activity to assess the change in the neuromuscular function. Evaluation based on functional tests does not directly involve a running task, and, thus, distinction between evaluation on treadmill and overground is irrelevant for this parameter group. Countermovement jump (CMJ) height and isometric MVC knee force were the most frequently studied parameters, both being analyzed in seven studies and showing a clear decrease because of acute fatigue. Moreover, CMJ height showed the same behavior for moderate and intermittent fatigue, thus showing a consistent behavior across different fatigue protocols. DJ stabilization time was only studied for severe fatigue, and it presented a clear increase. The balance-related parameter, center of pressure (CoP) velocity, presented a clear increase because of intermittent and severe intensity protocols.

Metrics from walking as a functional test were obtained from one study with 200 participants and only for moderate fatigue. Contact time, peak pressure, total foot contact area, and forefoot loading impulse showed a clear decrease. Sprint completion time was measured by three studies and showed a clear increase, i.e., worse sprint performance, after intermittent fatigue. The MA parameters were found to be assessed only in studies with severe and heavy intensity protocols. Of these, only integrated EMG signal (iEMG) calf and iEMG hamstring presented a clear decrease. The other parameters (iEMG and RMS) presented non-significant changes or non-consistent trends (Table 2).

## Discussion

### Response to Fatigue

#### Influence of Exercise Intensity

The exercise intensity can modulate the response of the neuromuscular system and running biomechanics to acute fatigue. Indeed, stride length, impact force–time integral, peak tibial acceleration, max knee flexion angle during swing, and knee flexion/extension ROM during stance present different trends for different fatigue protocols when controlled for the running environment. Aerobic metabolism mainly fulfills the energy requirement in the moderate and heavy protocols and a combination of aerobic and anaerobic metabolism in severe and intermittent protocols (Morton, 2006). It has been suggested that short-term high intensity activities mainly lead to peripheral fatigue (Perrey et al., 2010), whereas high volume activities, especially prolonged running, can lead to central fatigue (Millet and Lepers, 2004) in addition to structural and metabolic modifications. These mechanisms can potentially explain the differences in the neuromuscular response between different fatigue protocols (Brownstein et al., 2021). While there is a wealth of research (Laursen, 2010; Gibala et al., 2012) on long-term adaptation to various exercise intensities, we recommend further research to understand the mechanisms leading to differences in short-term responses.

Some parameters, despite the differences between the running environment and the fatigue intensity, presented a consistent response to acute fatigue. Cadence, contact time, flight time, peak tibial acceleration, trunk flexion, angle and knee flexion angle at IC increased because of fatigue, not necessarily by the same relative magnitude. Similarly, max GRF, and vertical and leg stiffness responded to acute fatigue by showing a decrease in magnitude. The trends for GRF, knee, and trunk kinematics are in line with a previous review (Winter et al., 2017) on the effect of fatigue due to prolonged running, which had a study sample of 12. This study computed the trends not only for prolonged running (Winter et al., 2017) but also for shorter, more intense running and interval running. Therefore, it allows for the comparison of parameter trends across different protocols and exercise intensities, highlighting the differences and similarities between responses to different conditions.

#### Acute Fatigue Affects Impact Load Attenuation and Leads to Sub-optimal Running Technique

Calf muscles play a crucial role in regulating the stiffness of muscle–tendon units to tolerate and absorb high impact loads at the beginning of the ground contact and the braking phase (Kyröläinen et al., 2005; Rabita et al., 2013). Acute fatigue leads to a lowered pre-activation in calf muscles, as evidenced by the decreasing trend for iEMG (Table 2). This hampers the ability of the musculoskeletal system to absorb the energy from impact, sustain impact loads, and return stored elastic energy in a coordinated manner during push-off (Avela et al., 1998). Reduced absorption of impact forces is likely to explain (Sheerin et al., 2019) the observed clear increase in peak tibial acceleration during the initial phase of ground contact (Voloshin et al., 1998). The increase in knee flexion angle during initial contact, linked to a lowered vertical stiffness (Table 2), might be an alternative attenuation strategy, an adaptation to overcome neuromuscular deficits. Another possible adaptation might be the increase in the relative proportion of ground contact time, thus distributing the impact impulse over a longer duration and reducing peak impact forces (Strohrmann et al., 2012). Peak impact forces can be a risk factor for bone stress injury (Hreljac, 2004; Warden et al., 2014; Davis et al., 2016), thus highlighting the importance of this result for injury prevention.

Forward leaning, as well as the variability of trunk movements, increase with fatigue of the lower back muscles (Table 2). This might increase injury risk by increasing the strain on the hamstrings and the back during running (Koblbauer et al., 2014; Maas et al., 2018). However, prior research has also suggested that increased trunk flexion during running might be a compensatory strategy for shock attenuation (Saha et al., 2008). Further investigations should consider the relationship between running kinematics and core stability, their causality, and to what extent these relations affect performance and injury risk.

The observation that acute fatigue leads to a decrease in vertical max GRF (Table 2) can be linked to a series of kinematics, kinetics, and muscular adaptations throughout fatiguing activities. The observed rise in contact time, in accordance with muscle fatigue, indicates that runners are not able to lift their feet off the ground as fast as before. Consequently, the push-off force is distributed over a longer duration, with a decrease in the max GRF (Winter et al., 2017). This decrease in the maximal force production capacity of the lower limb muscles during the push-off phase is confirmed by the decrease in the generated force during knee extension movements within the MVC tests. Increased knee and trunk flexion/extension angles, along with a reduction in vertical stiffness, point to an increased vertical motion of the center of mass (COM) due to acute fatigue. According to the spring-mass model, a decrease in vertical stiffness is consistent with the decrease in max GRF and an increase in the vertical displacement of the COM caused by the rise in maximum knee and hip flexion/extension angles and ROM. These trends are confirmed by the results (Table 2), and they support the rationale for increased vertical motion of the COM because of acute fatigue.

Energy efficiency during running is maintained partly by the elastic structures (tendons and muscles) in lower limbs, through the storage and return of elastic potential energy generated from the impact with ground (Novacheck, 1998). The lowered calf muscle activity, increased peak tibial acceleration (PTA), and the vertical displacement of COM indicate an increased transfer of the impact energy to the COM of the body and a reduction in the elastic potential energy absorbed from impact. Furthermore, a major source of energy loss (Bertram and Hasaneini, 2013) in running is the transition of the body motion from downward to upward direction in each gait cycle. An increase in this vertical motion of the COM, thus, points toward a lowered energy efficiency in running gait and a suboptimal running technique. However, it is difficult to ascertain whether the changes in running biomechanics originate from a strategy to protect against injuries or represent a fatigue-induced loss of optimal performance capabilities, or a combination of both.

#### Role of Functional Tests

CMJ tests typically measure the capacity of the leg extensor muscles to generate mechanical power (Schmitz et al., 2014), whereas MVC tests (Peñailillo et al., 2013) measure the capacity of leg muscles to exert their maximum force against resistive apparatus. Results (Table 2) show a decreased hamstring and calf muscles activation, also indicated by the decreased MVC force and increased sprint completion times. This can be explained by neuromuscular alterations, which provoke a slower rate of muscle force production possibly *via* slower recruitment of motor units.

To date, most research on sport-induced fatigue has been focused on the acute physiological and neuromuscular responses. As indicated in (Degache et al., 2014), postural control is a permanent re-establishment process of balance, which depends on the orientation information derived from the somatosensory, vestibular, and visual input sensory sources. Based on relevant postural muscles, the central nervous system actively controls balance. The results (Table 2) show that acute fatigue affects balance, underlined by the consistent increase in the balance parameters such as CoP velocity, LDS, and stabilization time in DJ. These results are consistent with the observation (Nardone et al., 1997) that participating in exhaustive physical activities can lead to a deterioration of the proprioceptive sensory information or its integration, thereby adversely affecting the efficiency of the neuromuscular system.

### Influence of Protocols

Treadmill running biomechanics may differ from overground running (Sinclair et al., 2013; Van Hooren et al., 2020) during the foot strike, in terms of peak propulsive force and sagittal plane joint kinematics like hip flexion/extension angles and ROM, knee flexion angle and ROM, foot strike angle, and COM vertical displacement. While debated (Van Hooren et al., 2020), some studies also indicate differences in muscle activity, impact peak GRF, and tibial forces (Milgrom et al., 2003; Kluitenberg et al., 2012; Baur et al., 2018). To investigate if these differences modulate the influence of fatigue on running, we computed summary trends for the treadmill and overground running studies separately. For the same exercise intensity, the two running environments led to different trends (Table 2) for stride length, peak impact pressure, and impact force-time integral. Thus, there is a considerable interaction between fatigue and type of running ambulation (i.e., treadmill or overground) for parameters directly related to foot strike, in accordance with the results from Strohrmann et al. (2012) and Garcia-Perez et al. (2014).

Fatigue typically leads to a reduction in speed while running overground (Bertram and Hasaneini, 2013), indicated by increased sprint completion time in a fatigued state and a simultaneous decrease in cadence and stride length (Table 2) for moderate-intensity acute fatigue. While professional athletes tend to modulate their pace tactically while running overground in competitions (Dierks et al., 2010), studies in this review typically used constant speed exercises on treadmills to analyze the effects of fatigue. Running speed has a direct influence on spatiotemporal parameters (Bailey et al., 2018a), and forcing a specific treadmill speed prevents fatigued athletes from modulating their running mechanics naturally. While non-motorized treadmills allow the athletes to run at self-selected speeds, they can lead to an increased metabolic demand as compared with overground running at the same speed (Edwards et al., 2017); higher metabolic demands can accelerate the development of fatigue. Furthermore, compliance of the running surfaces can affect the ground contact time, step length, plantar loading, and metabolic cost of running (McMahon and Greene, 1979; Smith et al., 2016), thus highlighting the critical nature of the running surface while testing.

### Recommendations for Using an IMU-Based Wearable Sensor Setup

As seen in earlier section, there is a difference between the results for treadmill and overground running in a fatigued state, especially due to the alternations in speed caused by fatigue while running overground. To improve the translatability of results, we argue for in-field monitoring of running mechanics. Furthermore, continuous measurement of biomechanics during the run can enable an understanding of the temporal evolution of the running technique in response to acute fatigue. If the alterations in running technique are too drastic and occur during several consecutive sessions, it can be an indication of poor adaptation to training. Wearable sensors allow for a continuous measurement during overground and treadmill running and across different real-world contexts such as outdoor training and competitive races. Wearable sensors can also allow the rehabilitation of runners suffering from running-related injuries, based on real-time feedback of running biomechanics and by combining the movement data with the applied training load (Willy, 2018). Early detection of such alterations using wearable sensors can be helpful to prevent adverse training adaptations.

Considering the importance of field measurement, some recommendations about the usage of wearable sensors may be helpful. The first step toward the design of an IMU-based wearable sensor setup is the selection of the parameters for measurement. Here, the parameters that showed consistent trends for the influence of acute fatigue (Table 2) could be a starting point. Among these parameters, sagittal plane knee angles and vertical GRF can be estimated with one sensor on the shank and one on the sacrum (Lee et al., 2010; Wouda et al., 2018); contact time, flight time, and vertical stiffness can be computed from either a sensor on the shank or the sacrum. However, foot-based IMUs and pressure insoles provide higher accuracy for the estimation of contact time and GRF (Falbriard et al., 2018). It is possible to estimate stride length as a combination of the running speed measured from a shank or an upper back sensor (Yang et al., 2011; Apte et al., 2020), and the cadence from a sacrum or foot sensor (Lee et al., 2010; Falbriard et al., 2018). While previous research has shown the measurement of sagittal hip angles to be possible for fast movements (Fasel et al., 2018), the accuracy of this measurement is susceptible to soft tissue artefacts.

**Figure 5:**
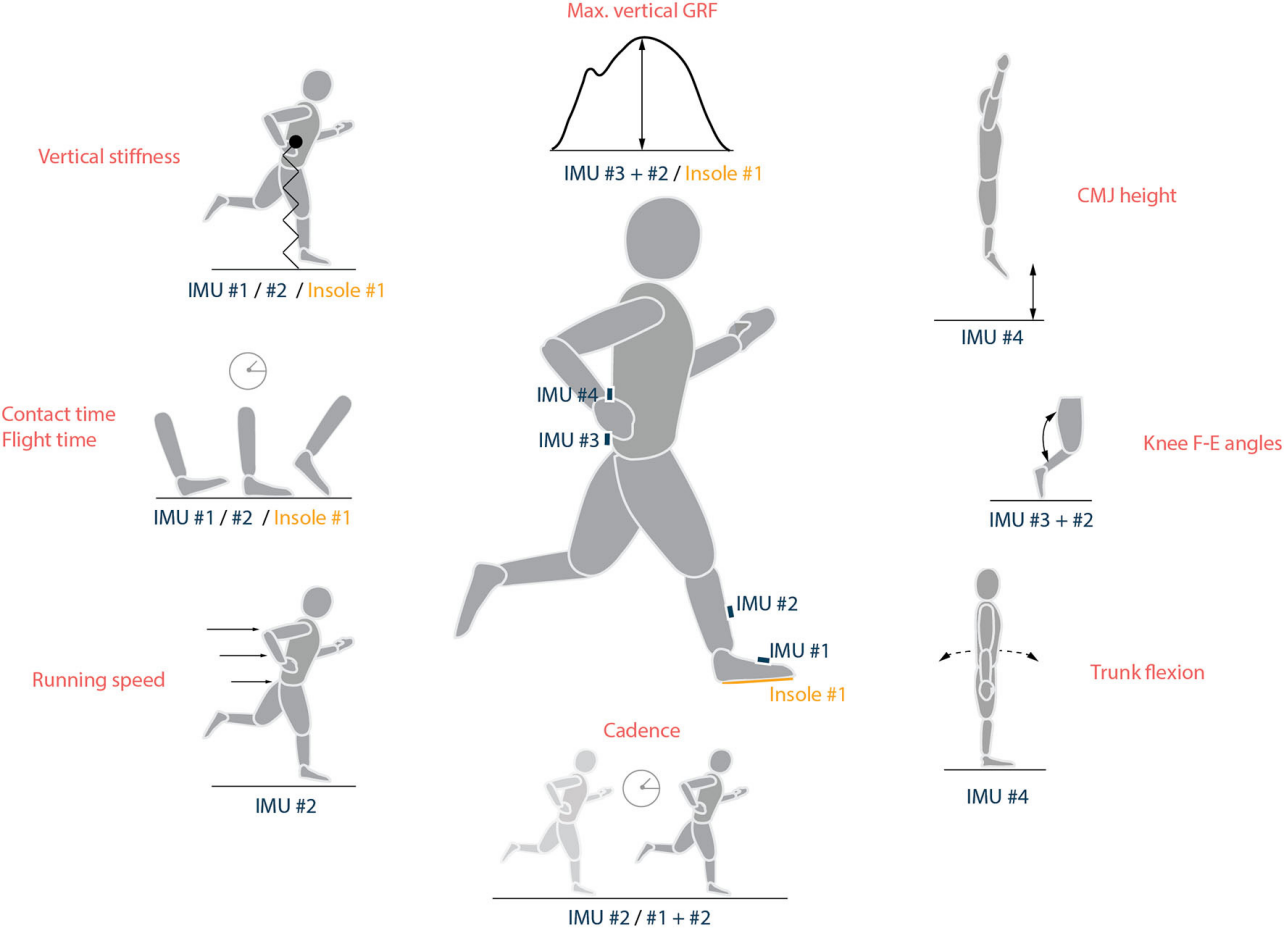
Parameters that show a consistent trend in response to acute fatigue and a potential wearable sensor setup to measure them. Stride length can be estimated by multiplying running speed and gait cycle time for each stride, while tibial acceleration can be measured directly from IMU#2. IMU, inertial measurement unit.

Apart from these biomechanical parameters in running, a single IMU located on the lumbar spine (L1) has been used for the assessment of vertical jump height (Setuain et al., 2016) and postural control ability (Neville et al., 2015). Thus, a minimal sensor configuration (Figure 5) based on only three or four sensors, one unit on the shank, one on the sacrum, one on lumbar spine (L1), and optionally one IMU on the foot or pressure insoles, could enable the measurement of the evolution of biomechanical parameters in response to acute fatigue. The sensors on shank and foot can be placed on both legs if the goal is also to investigate symmetry. Previous studies on this topic have either focused on the biomechanics of the whole body (Strohrmann et al., 2012; Op De Beeck et al., 2018) or a specific body segment (Voloshin et al., 1998; Mizrahi et al., 2000a; Derrick et al., 2002; Clansey et al., 2012; Garcia-Perez et al., 2014), thereby limiting the outcomes or being cumbersome to replicate. The suggested configuration offers a good balance between the number of sensors and the possibility to study a broad range of parameters that present a reliable response to acute fatigue. Algorithm development in the future might reduce the number of requisite sensors to only one IMU on the trunk (sacrum or L1).

For the measurement using wearable sensors, we recommend a static period of few seconds at the start of the run to facilitate the calibration of the sensors. The sampling rate (SR) of the used sensors should be set according to the movement of interest. For example, an SR of at least 500 Hz is recommended for measuring impact acceleration at the heel and other kinetic parameters at the foot, while a minimum SR of 333 Hz is suggested for estimating step length and 200 Hz for kinematic parameters, stride duration, and tibial acceleration (Mitschke et al., 2017). An SR of 1,000 Hz should suffice for almost all scenarios except sprinting, where a higher SR might be necessary for accurately estimating the impact forces at the foot (Mitschke et al., 2017). A lower-than-appropriate SR leads to inaccuracy in estimation, while an excessive sampling rate places a high demand on the battery and the storage of the sensors. For improved accuracy of measurements, it is essential to ensure correct fixation of the sensors to reduce undesired vibrations due to the impact of the foot on the ground. In case of repeated measurements, it is important to recheck the sensor fixation in order to detect any loosening and avoid undesirable movement of the sensors. As the algorithms (Falbriard et al., 2018; Wouda et al., 2018) typically work as desired at different speeds, the protocol can involve either fixed speeds or self-selected speeds. However, around 10 gait cycles at relatively stable speed will provide a more reliable estimate of the gait parameters for a given time period (Falbriard, 2020).

### On Study Protocols

The quality of the studies was scored based on the MINORS scale designed for non-randomized studies. The two criteria with usually the lowest scores are the inclusion of consecutive participants and the prospective calculation of study size. Only 12 studies tested more than 30 subjects (17.5%), with males as the large majority (66%). Considering the high inter-subject variabilities in terms of morphology and running techniques, a higher sample size could help improve interpretations of obtained parameter trends by making subgroups. Moreover, few studies compared amateur and professional athletes; male and female, or exercise intensities (intermittent vs. continuous). A higher number of comparative studies would improve the specificity of the results.

As seen in the results section, the most commonly used protocol to induce fatigue was treadmill running until exhaustion, classified as severe. Even in this very specific fatiguing activity, there is no agreement in the literature about which reference metric should be used for measuring fatigue. Several studies used questionnaires (RPE), while others used speed thresholds, VO_2_ max tests, heart rate zones, or a combination of those metrics. This lack of agreement makes it difficult to compare different protocols and explains certain inconsistencies across studies within the four subgroups for fatigue.

Finally, this systematic review allows us to highlight the current gaps in literature regarding sport-induced fatigue. One of the main findings is the lack of field studies with continuous measurements, conducted during the actual run. As seen in the results (Section Nature of Measurement Environment), stationary measurement systems represent 76.5% of sensors used, significantly more than wearables; and the ratio between stationary vs. wearable motion sensor has not changed over time (Figure 4). The main reason is that studies performed in-laboratory allow for highly controlled environmental conditions and are generally easier to perform. However, the recent burgeoning market of wearables, miniaturization of sensors, and development of advanced algorithms (Camomilla et al., 2018) have given researchers the capability to collect and analyze continuous data during sporting activates with good accuracy and precision.

### Limitations of This Review

The first limitation of this study is that studies involving different athlete groups with varying skill levels (elite athletes vs. amateur) and physical capacity were analyzed together to create summary trends. Mixing different study populations might lead to confounding effects in the computation of trends. However, this was done to overcome the limited number of studies within each subgroup and ensure large enough sample size for computing meaningful summary trends. As a result, the trends produced from the analysis can be generalizable across a wide population.

The parameters for analysis were selected based on the threshold of at least 30 participants within a fatigue category and/or running surface. This threshold was chosen with the aim of balancing the strength of evidence and the number of analyzed parameters. While a higher threshold would increase the strength of evidence per parameter, the number of analyzed parameters would have been drastically reduced since the majority of studies had <20 participants. A small change in one of the parameters might be more pertinent to the biomechanical response than a large change in another parameter, which makes it difficult to decide the importance of parameters *a priori*. To account for this, and for the lack of a single metric to characterize biomechanical response, we decided to include a large number of parameters despite the relative lack of research for some of them.

## Conclusion

This review presents evidence that acute fatigue influences almost all the included biomechanical parameters in running, with crucial influence from the exercise intensity and the testing environment. In response to acute fatigue, flight time, contact time, knee flexion angle at initial contact, trunk flexion angle, peak tibial acceleration, CoP velocity during balance test showed an increasing trend and cadence, vertical stiffness, knee extension force during MVC, maximum vertical ground reaction forces, and CMJ height showed a decreasing trend across different fatigue protocols. The results reaffirm the observations that acute fatigue causes a reduction in the maximal force production of the muscles and adversely affects the postural control ability, leading to a more compliant leg and a decreased attenuation of the impact force during each ground contact. The dominant metrics used for fatigue analysis were gait spatiotemporal parameters, while stationary sensor systems, treadmill activities, and endpoint measurements were the dominant modalities. The results indicate an important research gap with the lack of field studies with continuous measurement, conducted during actual sporting activities. Emerging technologies like wearable sensors could enable the design of such protocols, thus leading to a deeper understanding of the influence of fatigue on the biomechanics of the lower extremities. An outcome of this review is the proposal of a wearable sensor configuration based on three or four sensors, which will enable continuous in-field measurement of metrics that show a reliable response to acute fatigue. The metrics identified here could be used for athlete monitoring and the design of optimal training regimens, leading to an enhanced performance improvement/injury risk prevention ratio.

## Data Availability Statement

The original contributions presented in the study are included in the article/Supplementary Material, further inquiries can be directed to the corresponding author/s.

## Author Contributions

GP and SA performed the systematic search, information extraction, data analysis, evidence synthesis, and wrote the first draft of the manuscript. GP, SA, AM, CS, and TS performed article screening, risk of bias assessment, and study selection, with each study being appraised by two authors. All authors contributed to the study design, discussion of the obtained data and results, final manuscript, reviewed the final manuscript, and assumed responsibility for the information presented therein.

## Conflict of Interest

The authors declare that the research was conducted in the absence of any commercial or financial relationships that could be construed as a potential conflict of interest.

## Publisher's Note

All claims expressed in this article are solely those of the authors and do not necessarily represent those of their affiliated organizations, or those of the publisher, the editors and the reviewers. Any product that may be evaluated in this article, or claim that may be made by its manufacturer, is not guaranteed or endorsed by the publisher.
