# Supplementary material for: Biomechanical Response of the Lower Extremity to Running-Induced Acute Fatigue: A Systematic Review
Source: Front Physiol. 2021 Aug 27;12:646042. doi: 10.3389/fphys.2021.646042 (PMC8430259; doi:10.3389/fphys.2021.646042)
Supplement: Supplementary file 3 [file Data_Sheet_3.docx]

# Supplementary Material

## A1. Eligibility criteria

Below are some additional exclusion criteria that are not mentioned in section 2.2 for the sake of brevity.
*Exclusion criteria:*

- The study that focused only on biochemical parameters such as lactate, creatine kinase, cortisol, etc. to assess the effect of sport-related fatigue.
- The study which focused only on questionnaires (Borg, fatigue…) to assess fatigue, without additional sensors used.
- The study which evaluated the effect of sport on mental health based on self-reported questionnaires (motivation, mood, depression, self-reported, burnout).
- The study that focused on recovery time or training program after fractures or injuries related to sport. We also excluded the studies investigating sport-related concussion.
- The study that analyzed the effects of certain therapy to reduce fatigue such as phototherapy, cold water immersion therapy, diet, acupuncture, compression garments, moxibustion, etc.
- The study that focused on the effects of specific environmental conditions on fatigue or for training such as extreme conditions, altitude training, hypobaric chamber, acclimatization, hypoxic condition, etc.
- The study that analyzed the effects of performance-enhancing substances such as ergogenic aids, Beta-alanine effects, doping, heat acclimation, metformin, ischemic preconditioning, β2-agonists, etc.
- Studies that focus only on feedbacks to athletes
- Studies that focus on chemical/brain stimulation and other invasive methods effects on fatigue.
- Studies that focus on recovery time after fracture/injuries related to sport, including but limited to concussion.
- Studies that involve a biopsy procedure
- Studies aimed at validation of functional tests and not the evaluation of the influence of fatigue
- Research that utilizes specific exercises to induce, but which are not sport activity. For example, repetitive shoulder movement or resistance training.
- Studies that focus only on the biomechanics of sports other than running
- Pregnancy-related guidelines on physical activity
- Studies on animals
- Research investigating the effects of age on fatigue, or based on adolescents (<18 years) or old (>65 years) participants
- Studies that focus on biochemical parameters only

## A2. Study appraisal

The items mentioned below, based on the MINOR scale, were used to appraise the selected studies. Example for one study is provided in Table 3; supplementary material S1 presents the same for all studies.

Table 1 Adapted MINOR scale used for scoring the studies, with score from 0 to 2 for each metric

|  | A clearly stated aim | Inclusion of consecutive participants | Clear description of measurement system | Endpoints appropriate to the aim of the study | Unbiased assessment of the study endpoint | Follow-up period appropriate to the aim of the study | Loss to follow up less than 5% | Prospective calculation of the study size: | Total |
| --- | --- | --- | --- | --- | --- | --- | --- | --- | --- |
| Rousanoglu2016JSportSciMed [126] | 2/2 | 1/2 | 2/2 | 2/2 | 0/2 | 2/2 | 2/2 | 1/2 | 12/16 |

## A3. Parameter definition

(1) *local dynamic stability (LDS):* LDS can be quantified by the determination of the largest Lyapunov exponent λ and is interpreted as the ability to compensate small perturbations to maintain functional locomotion [71]. A higher exponent indicates a lowered ability to maintain stability. (2) *Peak force:* it is the maximum force measured in different regions of the foot [33,36,45,62,64] using pressure insoles and as such, cannot be directly compared to the ground reaction forces as they represent the summation of forces in all foot regions. (4) iEMG: Area under the curve of the rectified EMG signal i.e. the mathematical integral of the absolute value of the raw EMG signal [39,45,83] (5) MF: Median frequency of the EMG power spectrum [19,39,86,114,115]

## A4. Data synthesis


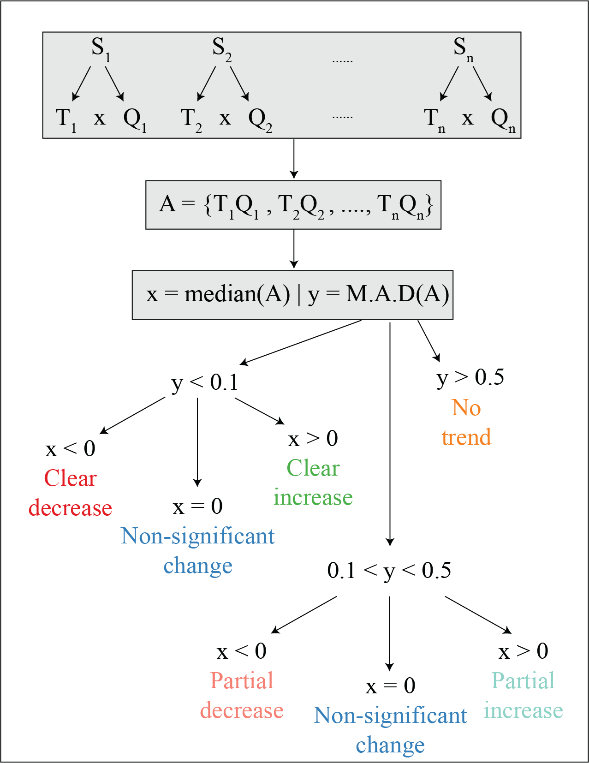


Figure 1 Flowchart for the extraction of the summary trends for different parameters, where S: study, T: trend, Q: study quality, and MAD: median absolute deviation. This process is followed for every parameter within each fatigue category.

The flowchart for the computation of summary trends for the parameters is shown in Figure 6; a process followed for every parameter within each subgroup. For each study (Si), we extracted quality score (Q_i_) and a list of parameters used to assess fatigue with their respective trend (T{j,i}, j: #parameter, i: #study). The parameter trends (T{j,i}) were multiplied by the respective study quality score (Q_i_) and combined into a vector (A_i_). Then, the median (MED) and the median absolute deviation (MAD) of A_i_, which correspond to the median and M.A.D of the different trends obtained for this parameter (T_j_), were computed. If MAD(A_i_) was greater than 0.5, we consider that there is no trend, i.e. no consistencies across studies. MAD(A_i_) lower than 0.1, indicated agreement across studies and was characterized as *“clear decrease”* if MED(A_i_) was negative, *“non-significant change”* if MED(A_i_) equal to zero, and *“clear increase”* if MED(A_i_) was positive. For 0.1<MAD(A_i_)<0.5 the trends were characterized as *“partial decrease”*, *“non-significant change”* or *“partial increase”* respectively.
